# Vasodilatory Effect of the Stable Vasoactive Intestinal Peptide Analog RO 25-1553 in Murine and Rat Lungs

**DOI:** 10.1371/journal.pone.0075861

**Published:** 2013-09-19

**Authors:** Jun Yin, Liming Wang, Ning Yin, Arata Tabuchi, Hermann Kuppe, Gerhard Wolff, Wolfgang M. Kuebler

**Affiliations:** 1 Department of Cardiothoracic Surgery, Affiliated People's Hospital of Jiangsu University, Zhenjiang, Jiangsu, China; 2 The Keenan Research Centre of the Li Ka Shing Knowledge Institute, St. Michael's Hospital, Toronto, Ontario, Canada; 3 Institute for Anesthesiology, German Heart Institute, Berlin, Berlin, Germany; 4 Institute of Physiology, Charité-Universitätsmedizin Berlin, Berlin, Germany; 5 Department of Translational Medicine, Hoffmann-La Roche Ltd., Nutley, New York, United States of America; 6 Departments of Surgery and Physiology, University of Toronto, Ontario, Canada; Pulmonary Research Institute at LungClinic Grosshansdorf, United States of America

## Abstract

**Rationale:**

Stable analogs of vasoactive intestinal peptide (VIP) have been proposed as novel line of therapy in chronic obstructive pulmonary disease (COPD) based on their bronchodilatory and anti-inflammatory effects. We speculated that VIP analogs may provide additional benefits in that they exert vasodilatory properties in the lung, and tested this hypothesis in both *ex vivo* and *in vivo* models.

**Methods:**

In isolated perfused mouse lungs and in an *in vivo* rat model, pulmonary blood vessels were preconstricted by hypoxia and hemodynamic changes in response to systemic (*ex vivo*) or inhaled (*in vivo*) administration of the cyclic VIP analog RO 25-1553 were determined.

**Results:**

In mouse lungs, RO 25-1553 reduced intrinsic vascular resistance at normoxia, and attenuated the increase in pulmonary artery pressure in response to acute hypoxia. Consistently, inhalation of RO 25-1553 (1 mg·mL^−1^ for 3 min) caused an extensive and sustained (> 60 min) inhibition of the pulmonary arterial pressure increase in response to hypoxia *in vivo* that was comparable to the effects of inhaled sildenafil. This effect was not attributable to systemic cardiovascular effects of RO 25-1553, but to a lung specific reduction in pulmonary vascular resistance, while cardiac output and systemic arterial hemodynamics remained unaffected. No adverse effects of RO 25-1553 inhalation on pulmonary gas exchange, ventilation-perfusion matching, or lung fluid content were detected.

**Conclusion:**

Our findings demonstrate that inhaled delivery of the stable VIP analog RO 25-1553 induces a potent and sustained vasodilatory effect in the pulmonary circulation with no detectable adverse effects. Therapeutic inhalation of RO 25-1553 may provide vascular benefits in addition to its reported anti-inflammatory and bronchodilatory effects in COPD, yet caution is warranted given the overall poor results of vasodilator therapies for pulmonary hypertension secondary to COPD in a series of recent clinical trials.

## Introduction

Projections for 2020 indicate that chronic obstructive pulmonary disease (COPD) will become the third leading cause of death worldwide in comparison to ranking 6^th^ in 1990 and fifth leading cause of years lost through early mortality or handicap (disability-adjusted life years) as compared to ranking 12^th^ in 1990 [1]. Yet, the rapidly increasing incidence and the associated socioeconomic burden on public health systems are contrasted by the current lack of effective therapeutic options for prevention or therapy of this disease.

Vasoactive intestinal peptide (VIP) is a vasodilatory peptide that was first isolated from the upper intestine [2] and that exerts prominent smooth muscle relaxant as well as anti-inflammatory and immunomodulatory properties [3]. VIP is abundantly present in normal human lungs, including tracheobronchial smooth muscle cells, glands of airways, and pulmonary vascular walls [4], [5]. The biological actions of VIP are mediated by two type II G-protein coupled receptors, VIP/pituary adenylate cyclase-activating polypeptide type I (VPAC1) and type II (VPAC2) [6], which are expressed on airway epithelia, macrophages, and in pulmonary arteries and veins [7], [8]. Recently, VPAC agonists such as VIP and synthetic analogs thereof have emerged as promising novel line of therapy for the treatment of obstructive and inflammatory airway disease such as COPD. As compared to VIP, the second generation VIP analog RO 25-1553 [9], [10] and the chemically related follow up molecule RO 50-24118 [11] are biologically more stable, and constitute potent and selective agonists of VPAC2. RO 50-24118 has been shown to have dual bronchodilatory and anti-inflammatory effects, in that it relaxes airway smooth muscle cells, inhibits bronchoconstriction and attenuates the influx of neutrophils and CD8^+^ T-cells in inflammatory lung disease [12].

On the vascular side, VIP or its natural analogs have previously been shown to relax isolated pulmonary artery segments, to antagonize pulmonary vasoconstriction, and to inhibit the proliferation of pulmonary vascular smooth muscle cells from patients with idiopathic pulmonary arterial hypertension [12], [13]. However, these hemodynamic effects are generally short-lived within the range of a few minutes due to the short half-live of VIP *in vivo* [14]. Based on the reported pulmonary vascular effects of VIP, we hypothesized that stable VIP analogs may exert beneficial effects in COPD patients that exceed their demonstrated bronchorelaxant and anti-inflammatory action. Specifically, stable VIP analogs may concomitantly exert pulmonary vasodilatory effects, provided that they demonstrate a similar yet more prolonged vasorelaxant profile as natural VIP. Such a dual-pronged bronchial and vascular action would be particularly attractive in light of the fact that pulmonary hypertension (PH) is prevalent in 30-50% of patients with advanced COPD [15], [16]. Presence of PH has been proposed to have significant clinical implications in advanced COPD [17] as it is associated with functional impairment and an increased mortality risk [15], yet caution is warranted not to mistake such association as proof of a necessary cause-effect relationship. Notably, analyses of data from the ASPIRE (Assessing the Spectrum of Pulmonary Hypertension Identified at a Referral Centre) registry did not detect an association of compassionate therapeutic targeting of the pulmonary vasculature with a survival benefit in COPD patients with severe PH; however, a subset of patients who responded to pulmonary vascular treatment either by an improvement in WHO functional class or by a fall in PVR >20% showed an increased cumulative survival as compared to non-responders [18]. While these findings suggest that targeting PH may be of potential clinical benefit in at least a subset of responders, recent clinical trials demonstrate that systemic delivery of vasodilators such as sildenafil or bosentan not only failed to show clinical improvement but frequently further aggravated arterial hypoxemia in COPD patients with PH [19]–[22].

In the present study, we aimed to evaluate the vasodilatory potential of the synthetic, stable VIP analog RO 25-1553 in the pulmonary circulation. To this end, we first determined the dose-response of pulmonary vascular tone to systemically administered RO 25-1553 in the isolated perfused mouse lung preparation. Next, we probed in an *in vivo* rat model whether pulmonary vasodilation could similarly be achieved by inhalative delivery of nebulized RO 25-1553. In both models, RO 25-1553 consistently attenuated the pulmonary vasoconstrictive response to hypoxia without detectable adverse effects on systemic hemodynamic or pulmonary gas exchange parameters, indicating that therapeutic administration of VIP agonists may exert additional vasodilatory effects in COPD patients.

## Materials and Methods

### Animals

Male C57BL/6 mice of 20–30 g body weight (bw) and male Sprague-Dawley rats (350–400 g bw) were obtained from Charles River Laboratories (St. Constant, QC). All animals received care in accordance with the "Guide for the Care and Use of Laboratory Animals" (Institute of Laboratory Animal Resources, National Academy Press, Washington, DC 1996). The study was approved by the Animal Care Committee of St. Michaeĺs (ACC protocols #992 & #995).

### Vasorelaxation in isolated perfused mouse lungs

Isolated perfused mouse lungs were prepared as previously described [23]. In brief, mice were anesthetized by intraperitoneal injection of pentobarbital sodium (100 mg·kg^−1^ bw; Bimeda-MTC Animal Health Inc., Cambridge, ON) and placed in a 37°C water-jacketed chamber (Typ 839, Hugo-Sachs, March, Germany). After tracheostomy, volume-controlled ventilation (MiniVent 845, Hugo-Sachs) was initiated with a tidal volume of 10 mL/kg bw, 90 breaths/min and a positive end-expiratory pressure of 2 cmH_2_O, and mice were ventilated with a normoxic gas mixture of 21% O_2_, 5% CO_2_, and 74% N_2_ (Praxair, Mississauga, ON). Following a midsternal thoracotomy, 10 U of heparin were injected into the right ventricle for anticoagulation. Two cannulas of 1 mm inner diameter each were inserted into the pulmonary artery and the left atrium, respectively, and lungs were perfused by Hank’s Balanced Salt Solution containing 5% bovine serum albumin and 5% dextran (all Sigma-Aldrich, Oakville, ON) [24]. Sodium bicarbonate was added to adjust the perfusate pH to 7.4±0.02. After flushing the lungs with ≥10 mL, the perfusion circuit was closed and lungs were perfused at 37°C with a constant flow rate of 50 mL·kg bw^−1^·min^−1^ at a left atrial pressure (LAP) of 2 mmHg by a roller pump (Ismatec, Glattbrugg, Switzerland). Pulmonary arterial pressure (PAP) and LAP were continuously measured via saline-filled membrane pressure transducers (Hugo-Sachs) connected to side ports of the inflow and outflow cannulas, respectively. Pressure transducers were connected to a transbridge amplifier (Hugo-Sachs) and data were recorded at 150 Hz per channel (Data Translation 4.0; Data Translation GmbH, Bietigheim-Bissingen, Germany).

### Analysis of pulmonary vasoreactivity in isolated perfused mouse lungs

As pulmonary vascular tone is physiologically low, the pulmonary vasodilatory response of the test compound was assessed in terms of its ability to prevent hypoxic pulmonary vasoconstriction (HPV). The extent of HPV was quantified in isolated perfused mouse lungs as increase in PAP (ΔPAP) in response to hypoxia (1% O_2_, 5% CO_2_, and 94% N_2_; Praxair) and intrinsic pulmonary vascular resistance R_0_ was calculated from pressure-flow (P-Q) curves at normoxic and hypoxic ventilation as previously described [24]. To determine R_0_, lungs were perfused at either normoxia or hypoxia with flow rates of 25, 50, 75 and 100 mL·kg bw^−1^·min^−1^ in randomized order for 30 s each to generate a four-point pressure-flow curve. LAP was adjusted to 2 mmHg at each flow rate, and PAP was measured at the end of each step. P-Q curves generated under normoxic and hypoxic ventilation were analyzed by nonlinear regression analysis according to the distensible vessel model [25] by a least-square fit to the equation PAP  =  ([(1+α×LAP)^5^+5 α×R_0_×Q]^0.2^ – 1)/α where R_0_ is the intrinsic vascular resistance of the lung, i.e. the resistance that would exist if the lung vessels were at their respective diameter at zero vascular pressure, α is the vascular distensibility factor describing the relation between vessel diameter and pressure when the diameter is normalized to the diameter at zero pressure, and Q is the applied perfusate flow.

### Monitoring of pulmonary hemodynamics in the intact rat in vivo

Experiments were performed as previously described in detail [26], [27]. In brief, rats were anesthetized by intraperitoneal injection of a triple combination of medetomidine (0.5 mg·kg^−1^ bw, Domitor®, Dr. E. Graeub AG, Basel, Switzerland), fentanyl (0.05 mg·kg^−1^ bw, JanssenCilag, Neuss, Germany), and midazolam (5 mg·kg^−1^ bw, Dormicum®, Roche, Basel, Switzerland) [28]. Rats were placed in supine position on a thermostatically controlled electric heating blanket (Homeothemic Blanket Control Unit, Harvard Apparatus, March-Hugstetten, Germany) to maintain body temperature at 38°C. After tracheostomy, rats were mechanically ventilated with room air at a tidal volume of 6 ml·kg^−1^ bw, 110 breaths/min and a peak inspiratory pressure of 10.5±1 cmH_2_O. Via the left carotid artery and the right jugular vein, polyvinyl catheters with an internal diameter of 0.58 mm were inserted into the aorta and vena cava for measurement of arterial and central venous pressure as well as drug administration, respectively. Following a median thoracotomy the pericardium was opened and a catheter was introduced via the left auricle into the left atrium. A second catheter was advanced into the pulmonary artery via the right ventricle. An ultrasonic flow probe (Transonic®, Transonic Systems Inc., Ithaca, NY) was placed around the ascending aorta distal to the branching of the coronary arteries. Arterial pressure (AP), central venous pressure (CVP), PAP, LAP and aortic flow (AF) were continuously registered and digitally recorded. Pulmonary vascular resistance (PVR) was calculated as arteriovenous pressure difference over flow. Following post-surgical stabilization, baseline hemodynamic data were recorded over 5 min during normoxic ventilation (21% O_2_), and arterial and venous blood gas analyses were performed. For each blood gas analysis, the pulmonary shunt fraction (*Qs*/*Qt*) was calculated according to the classic shunt equation as *Qs*/*Qt* = (CcO_2_ – CaO_2_)/(CcO_2_ – CvO_2_) with CaO_2_, CcO_2_, and CvO_2_ reflecting O_2_ content in arterial, capillary, and venous blood. CcO_2_ was calculated from alveolar O_2_ partial pressure (PAO_2_) which was estimated based on the alveolar gas equation [29]. Ventilation was then either switched to a hypoxic gas mixture containing 11% O_2_, balance N_2_ (Messer Griesheim GmbH, Ludwigshafen, Germany) or continued at normoxia, and hemodynamics were again recorded after 5 min, followed by arterial and venous blood gas analyses. According to the randomized protocol, the appropriate test compound was inhaled for 3 min and hemodynamic data were again recorded after 5, 30 and 60 min. At the end of the protocol, a third arterial and venous blood gas analysis was performed and animals were euthanized by exsanguinations. For determination of lung edema, lungs were removed immediately *post mortem*, weighed, dried in a microwave oven for 40 min as described [30], re-weighed, and wet-to-dry lung weight ratio was calculated.

### Experimental groups

All drugs were dissolved in 0.9% saline as vehicle. *Isolated perfused mouse lungs*. After preparation, isolated perfused lungs were randomly assigned to one of five experimental groups (n = 8 animals each). Lungs in group I did not receive any vasodilator treatment (control). In groups II-IV, isolated lungs were perfused with the stable VPAC2 agonist RO 25-1553 (kindly provided by Hoffmann-LaRoche Inc., Nutley, NJ and produced as previously described) [9] at final concentrations of 0.01, 0.1 and 1 mg·ml^−1^, respectively. In group V, lungs were perfused with 100 nmol·l^−1^ sildenafil (Pfizer Inc.) as positive control [31]. *In vivo rat hemodynamics*. After completion of surgical preparation, animals were randomly assigned to one of six experimental groups (n = 8 animals each). Following baseline hemodynamic recordings, groups 1, 3 and 5 continued on normoxic ventilation, while groups 2, 4 and 6 were switched to hypoxic ventilation. After a second hemodynamic recording, groups 1 & 2 inhaled 0.9% NaCl solution (vehicle control), groups 3 & 4 the test compound RO 25-1553 (1 mg·ml^−1^), and groups 5 & 6 inhaled sildenafil (10 mg·ml^−1^) as positive control [32]. Each drug was nebulized using an ultrasonic nebulizer (Optineb®, Nebu-Tec, Elsenfeld, Germany) and inhaled for 3 min at identical peak inspiratory pressures as used throughout the experiment [26], [27].

### Statistical analyses

All data are presented as means ± SEMs. Treatment groups were compared by Mann-Whitney *U*-test or Kruskal-Wallis test. Normoxia and hypoxia data from the same mouse lung were compared by Wilcoxon matched pairs signed rank test. Dose-response curve was generated by non-linear regression analyses and fitted to a 3-parametric hyperbolic decay curve using SigmaPlot software (V9.0, Systat Software, San Jose, CA). Statistical significance was assumed at p<0.05.

## Results

### RO 25-1553 attenuates pulmonary vasoconstriction in isolated perfused lungs

**Figure 1:**
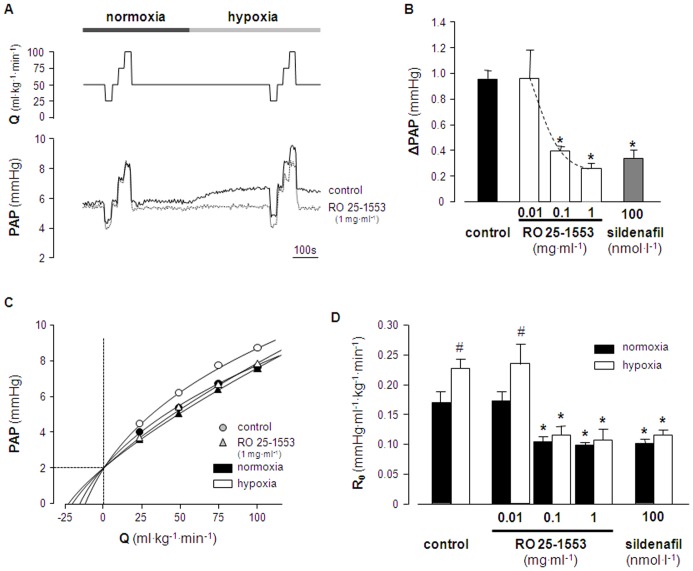
RO 25-1553 attenuates HPV in isolated murine lungs. **A**) Representative tracings of pulmonary arterial pressure (PAP; lower panel) in isolated mouse lungs perfused in the absence (control) or presence of RO 25-1553 (1 mg·mL^−1^) during stepwise changes in lung perfusion (Q; upper panel) at normoxia (21% O2) or hypoxia (1% O2). **B**) Group data showing the increase in pulmonary artery pressure (ΔPAP) relative to baseline measured 10 min after the change from normoxic (21% O_2_) to hypoxic (1% O_2_) ventilation in isolated mouse lungs perfused with buffer alone (*control*), with RO 25-1553 at final concentrations of 0.01, 0.1 or 1 mg·mL^−1^, and in lungs perfused with sildenafil (100 nMol·L^−1^) as positive control. **C**) Non-linear regression analysis according to the distensible vessel model yields representative pressure-flow (P-Q) curves for lungs perfused in the absence (control) or presence of RO 25-1553 (1 mg·mL^−1^) at normoxia and hypoxia. The pressure at Q = 0 ml·kg^−1^·min^−1^ reflects the LAP which was set to 2 mmHg, while the corresponding slope of the P-Q curve reflects R_0_. **D**) Group data showing intrinsic vascular resistance R_0_ at normoxia (21% O_2_) or hypoxia (1% O_2_) in isolated mouse lungs perfused with buffer alone (*control*), with RO 25-1553 at final concentrations of 0.01, 0.1 or 1 mg·mL^−1^, and in lungs perfused with sildenafil (100 nMol·L^−1^) as positive control. Group data are means±SEMs; * p<0.05 vs. control, # p<0.05 vs. normoxia, n = 8 experiments each.

In a first set of experiments, we tested whether RO 25-1553 can attenuate HPV in isolated murine lungs. Basal PAP in isolated mouse lungs was 5.6±0.1 mmHg and did not differ significantly between experimental groups. Single pressure tracings given in Fig. 1A show characteristic changes in pulmonary arterial pressure (PAP) during variations in perfusate flow (Q). After switching from normoxic to hypoxic ventilation, PAP increased in control lungs, yet the magnitude of the HPV response was substantially reduced in the presence of 1 mg·ml^−1^ of RO 25-1553. Group data analyses substantiated a distinct increase in pulmonary artery pressure (ΔPAP) during hypoxic ventilation in control lungs that was largely attenuated in lungs perfused with 0.1 or 1 mg·ml^−1^ of RO 25-1553 (Fig. 1B). This inhibition was comparable to the effect of the phosphodiesterase 5 inhibitor sildenafil (100 nmol·l^−1^) which served as positive control. Lower concentrations of RO 25-1553 (0.01 mg·ml^−1^), however, failed to inhibit HPV. The resulting dose-response for the inhibitory effect of RO 25-1553 on HPV could be described by a hyperbolic decay curve (Fig. 1B).

Analysis of pressure-flow curves was performed to obtain R_0_ as a pressure-independent measurement of vascular resistance. Representative raw P-Q relationships from individual experiments and calculated corresponding R_0_ values are given in Table 1, and resulting non-linear P-Q curves are shown for control and RO 25-1553 (1 mg· ml^−1^) perfused lungs at normoxia and hypoxia in Fig. 1C. R_0_, which is reflected by the slope of the P-Q curve at its intercept with the abscissa, increased with hypoxia in control lungs, whereas perfusion with RO 25-1553 resulted in markedly lower R_0_ values both at normoxia and hypoxia. Group analysis of calculated R_0_ data confirmed the inhibition of HPV by RO 25-1553 at concentrations of 0.1 and 1 mg· ml^−1^, as well as by sildenafil, yet not at the lowest concentration of RO 25-1553 (0.01 mg·ml^−1^; Fig. 1D). In addition, R_0_ analyses revealed a reduction in intrinsic pulmonary vascular resistance at normoxia by 0.1 and 1 mg·ml^−1^ RO 25-1553 as well as by sildenafil that was not immediately evident from the original PAP values alone (5.8±0.2 mmHg in controls versus 5.3±0.2 mmHg at both 0.1 and 1 mg·ml^−1^ RO 25-1553; no significant difference between groups). This finding demonstrates that RO 25-1553 did not only counteract pulmonary vasoconstriction, but also directly stimulated pulmonary vasodilation.

**Table 1:** Representative raw data for the pressure-flow (P-Q) relationships in isolated perfused mouse lungs at different experimental conditions.

group		control		RO 25-1553						sildenafil	
concentration		-		0.01 mg·ml^−1^		0.1 mg·ml^−1^		1 mg·ml^−1^		100 nmol/l^−1^	
FiO_2_		0.21	0.01	0.21	0.01	0.21	0.01	0.21	0.01	0.21	0.01
PAP	25 ml·kg^−1^·min^−1^	4.38	4.91	4.27	4.57	3.82	3.94	3.95	4.02	3.80	3.93
PAP	50 ml·kg^−1^·min^−1^	5.85	6.77	5.88	6.83	5.69	6.12	5.52	5.85	5.56	5.94
PAP	75 ml·kg^−1^·min^−1^	7.32	8.40	7.12	8.13	7.14	7.56	6.97	7.24	6.71	7.17
PAP	100 ml·kg^−1^·min^−1^	8.32	9.47	8.16	9.06	8.22	8.58	8.22	8.49	7.85	8.36
R_0_		0.17	0.23	0.18	0.23	0.11	0.13	0.10	0.12	0.11	0.12

Representative pulmonary artery pressures (PAP; in mmHg) as recorded in individual isolated mouse lungs perfused at flow rates (Q) of 25, 50, 75, and 100 ml·kg^−1^·min^−1^ and corresponding intrinsic pulmonary vascular resistance values (R_0_; in mmHg·ml^−1^·kg^−1^·min^−1^) as calculated from the respective 4-point P-Q curve. Examples are given for normoxic (21% O_2_) or hypoxic (1% O_2_) lungs perfused with buffer alone (*control*), with RO 25-1553 at final concentrations of 0.01, 0.1 or 1 mg·mL^−1^, or with sildenafil (100 nMol·L^−1^). FiO_2_; fraction of inspiratory O_2_ (0.21 for normoxia; 0.01 for hypoxia).

### RO 25-1553 inhalation reduces hypoxic pulmonary vasoconstriction in vivo

**Figure 2:**
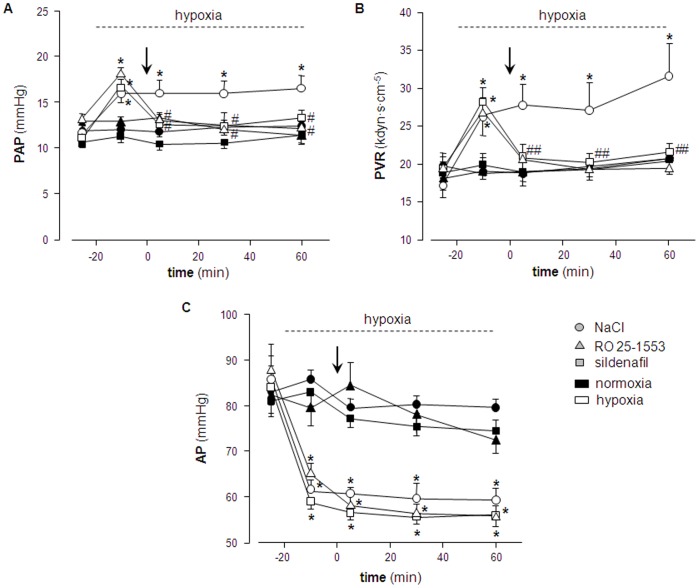
Inhaled RO 25-1553 attenuates HPV in the absence of systemic cardiovascular effects in vivo. Group data showing **A**) mean pulmonary artery pressure (*PAP*), **B**) pulmonary vascular resistance (*PVR*), and **C**) mean systemic arterial pressure (*AP*) in rats at normoxic baseline (t = –25 min), after switch to hypoxic ventilation (11% O_2_, *open symbols*) or during continued normoxic ventilation (21% O_2_, *black symbols*), respectively (t = –10 min), and 5, 30 and 60 min following a 3 min inhalation of either 0.9% NaCl (*circles*), RO 25-1553 (1 mg·mL^−1^; *triangles*) or sildenafil (10 mg·mL^−1^; *squares*). Interval of hypoxic ventilation (for hypoxia groups) is indicated by dashed line, time of drug inhalation (t = 0 min) by arrow. Lines connecting individual data points serve assignment to the different study groups and do not reflect exact time course of parameters. Data are means±SEMs; * p<0.05 vs. corresponding normoxia group, # p<0.05 vs. NaCl group under similar ventilation; n = 8 experiments each.

In a second set of experiments, we tested whether RO 25-1553 exerts a similar vasodilatory effect in the pulmonary vasculature when a) administered by inhalation and b) in an *in vivo* setting rather than an isolated organ preparation. In anesthetized and mechanically ventilated rats, a switch from normoxic (21% O_2_) to hypoxic (11% O_2_) ventilation caused a characteristic HPV response in form of a rapid and distinct increase in PAP that persisted over the remaining observation time of 60 min (Fig. 2A). Inhalation of either RO 25-1553 (1 mg·ml^−1^) or sildenafil (positive control; 10 mg·ml^−1^) reversed this increase in pulmonary vascular tone within < 5 min, while inhalation of 0.9% NaCl as vehicle control had no effect (Fig. 2A). Calculation of absolute PVR values demonstrated that the hypoxia-induced increase in PAP and its reversal by inhaled RO 25-1553 or sildenafil, respectively, were indeed attributable to changes in lung vascular resistance (Fig. 2B) rather than effects on cardiac output, which did not differ between groups over the course of the experiment (data no shown).

### RO 25-1553 inhalation had no detectable adverse effects in vivo

**Figure 3:**
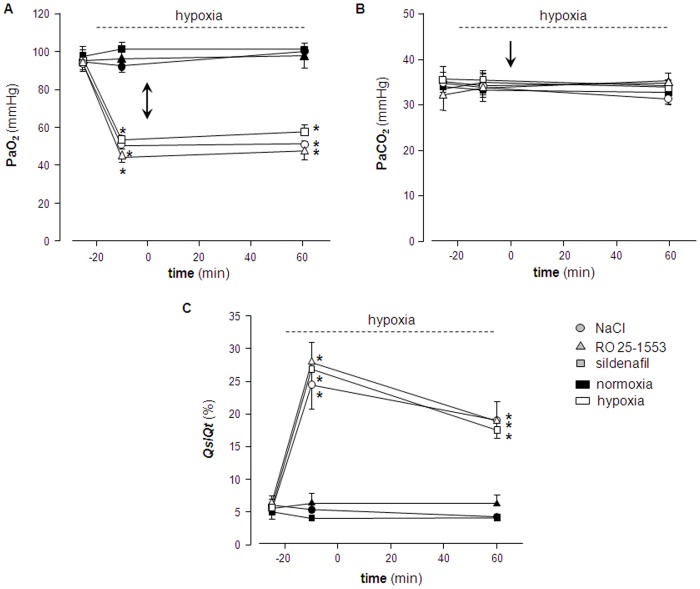
Inhaled RO 25-1553 does not impair gas exchange or ventilation-perfusion matching in vivo. Group data showing **A**) arterial partial pressure of O_2_ (*PaO_2_*), **B**) arterial partial pressure of CO_2_ (*PaCO_2_*), and **C**) pulmonary shunt fraction (*Qs/Qt*) in rats at normoxic baseline (t = –25 min), after switch to hypoxic ventilation (11% O_2_, *open symbols*) or during continued normoxic ventilation (21% O_2_, *black symbols*), respectively (t = –10 min), and 60 min following a 3 min inhalation of either 0.9% NaCl (*circles*), RO 25-1553 (1 mg·mL^−1^; *triangles*) or sildenafil (10 mg·mL^−1^; *squares*). Interval of hypoxic ventilation (for hypoxia groups) is indicated by dashed line, time of drug inhalation (t = 0 min) by arrow. Lines connecting individual data points serve assignment to the different study groups and do not reflect exact time course of parameters. Data are means±SEMs; * p<0.05 vs. corresponding normoxia group; n = 8 experiments each.

**Figure 4:**
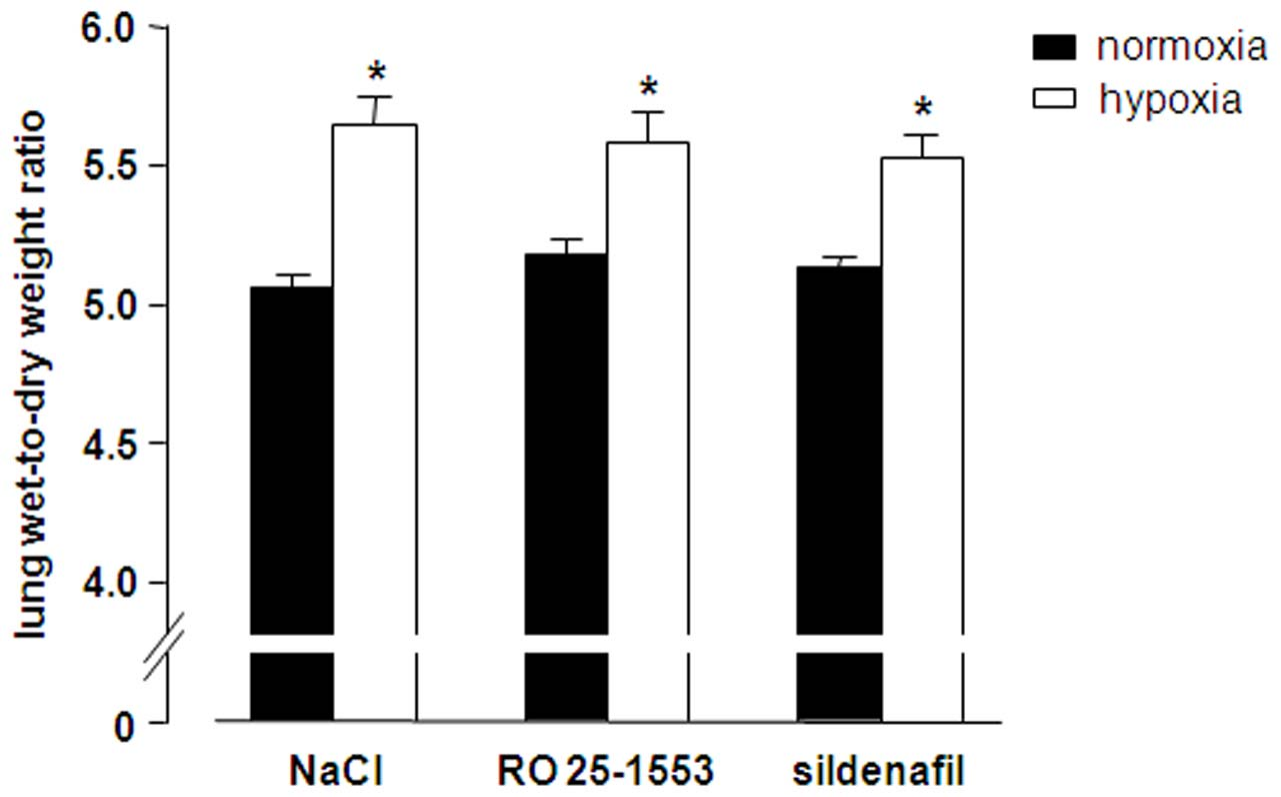
Inhaled RO 25-1553 does not increase lung water content in vivo. Group data showing lung wet-to-dry weight ratio as a measure of lung edema in rats after normoxic (21% O_2_, *black bars*) or hypoxic (11% O_2_, *open bars*) ventilation for 70 min and a 3 min inhalation of either 0.9% NaCl, RO 25-1553 (1 mg·mL^−1^) or sildenafil (10 mg·mL^−1^) 60 min prior to tissue harvesting. Data are means+SEMs; * p<0.05 vs. corresponding normoxia group, n = 8 experiments each.

In the same experiments, we next analyzed whether inhalation of RO 25-1553 may exert potential adverse effects on systemic hemodynamics, pulmonary gas exchange, ventilation-perfusion matching, or lung fluid balance. In parallel to the vasoconstrictive response in the pulmonary circulation, hypoxic ventilation caused a vasodilatory response in the systemic circulation as evident by the concomitant drop in systemic AP (Fig. 2C). Neither inhalation of RO 25-1553 nor of sildenafil had a detectable effect on AP in normoxic or hypoxic rats, indicating that the vasodilatory effect of the inhaled compounds did not spill over into the systemic circulation. In all experimental groups with hypoxic ventilation, arterial blood gas analyses revealed the expected arterial hypoxemia, yet arterial O_2_ partial pressure (PaO_2_) was not affected by inhalation of RO 25-1553 or sildenafil in either the normoxic or the hypoxic groups, respectively (Fig. 3A). Arterial CO_2_ partial pressure (PaCO_2_) remained unchanged in all groups over the course of the experiment (Fig. 3B). Similarly, RO 25-1553 did also not affect *Qs*/*Qt* in either normoxic or hypoxic lungs, indicating that the VPAC2 agonist did not interfere with ventilation/perfusion matching in the lung (Fig. 3C). Yet, in agreement with the well-described opening of intrapulmonary arterio-venous shunts by hypoxia [33], *Qs*/*Qt* increased significantly after switching to 11% O_2_ in all hypoxic groups. Concurrent with previous reports [34], hypoxic ventilation also caused mild pulmonary edema as indicated by a slight yet significant elevation in wet-to-dry lung weight ratios (Fig. 4). Notably however, neither inhalation of RO 25-1553 nor of sildenafil had a detectable effect on lung fluid content under these experimental conditions.

## Discussion

In the present study, we demonstrate the vasodilatory potential of the stable VIP analog RO 25-1553 as evident by its ability to reduce intrinsic vascular resistance in normoxic lungs, and to antagonize vasoconstriction in hypoxic lungs. RO 25-1553 proved effective in both an *ex vivo* mouse and an *in vivo* rat model when given either systemically or via inhalation, and its efficacy at systemic doses of 0.1 mg·ml^−1^ was comparable to that of the clinically established pulmonary vasodilator sildenafil (100 nmol·l^−1^). No adverse effects of inhaled RO 25-1553 on systemic hemodynamics, pulmonary gas exchange, ventilation/perfusion matching or lung edema formation were detectable. Hence, the effects of VPAC2 agonists in COPD may exceed their demonstrated dual bronchodilatory and anti-inflammatory potential, in that they concomitantly exert vasodilatory effects in the pulmonary circulation.

Despite extensive research efforts, COPD remains a major and still growing cause of morbidity, mortality, and socioeconomic health care burden worldwide that has not been matched by adequate and efficient therapies so far. Recent work has proposed VIP, one of the major peptide transmitters in the central and peripheral nervous system, as a promising agent for the treatment of chronic airway disease including asthma [35] and COPD [36], [37] based on its combined bronchodilatory and immunomodulatory properties. VIP is abundantly present in normal human lungs [38], along with its receptors, VPAC1 and VPAC2 [7], [8], [39]. Yet, the therapeutic applicability of VIP is thwarted by its cardiovascular side effects following systemic administration [40], and its rapid (<1 min) enzymatic degradation in a biological environment [41], [42]. To avoid systemic side effects, drug delivery by inhaled application of VIP or its analogs has been proposed [36] and has proven both effective and lung specific in the present study, in that inhalation of the VIP analog caused pulmonary, yet not systemic vasodilation *in vivo*. To overcome the rapid proteolytic cleavage of VIP, Bolin and coworkers developed RO 25-1553, a cyclic peptide analog of VIP that contains a lactam ring at the original primary cleavage site of the VIP molecule, resulting in higher potency, increased metabolic stability, and hence, a longer duration of action as compared to VIP [9], [43]. In a series of *in vitro* and *in vivo* models, RO 25-1553 has proven effective to attenuate airway hyperreactivity and inflammation [9], [10], [44], [45], and its maximum bronchodilatory effect during 24 hours was similar to that of the reference bronchodilator formoterol in a clinical study on 24 patients with moderate stable asthma [46].

Of relevance, genetic deletion of VIP in mice causes not only characteristic hallmarks of chronic airway disease including airway hyperresponsiveness and peribronchial inflammation, but also moderate pulmonary arterial hypertension and considerable pulmonary vascular remodelling [47], [48]. Along these lines, VIP has been found to relax human pulmonary vascular smooth muscle cells [49], to reverse leukotriene D_4_-induced contraction in isolated pulmonary arteries [50], and to counteract the pulmonary vasoconstrictor response to U46619 in cats [51]. A similar vasodilatory effect was also demonstrated for the VIP analog RO 25-1553 in isolated human pulmonary artery rings preconstricted with prostaglandin F_2α_ [52]. These findings generated considerable enthusiasm for the use of VIP and VIP analogs as novel therapeutic strategy in PH that was initially supported by a couple of single-center clinical trials which reported favorable effects of inhaled VIP in PH patients [53], [54]. A multicenter randomized controlled trial presented at the Annual Conference of the American Thoracic Society (ATS) 2010, however, found no reduction in PVR by VIP as compared to placebo either after single inhalation or after 12 weeks of treatment, although an exploratory analysis suggested some potential improvement in 6 minute walk test after 6 months of treatment [55]. With the publication of the final results from this study pending, the overall effectiveness of VIP and VIP analogs in PH remains to be resolved [56].

PH, defined as a mean pulmonary artery pressure exceeding 25 mmHg at rest, is also a common complication with a reported prevalence of 30–50% in patients with advanced COPD [15], [16]. PH secondary to COPD is classified in group 3 of the WHO classification of PH, i.e. PH associated with lung disease and/or hypoxia [57], and alveolar hypoxia is considered to be a key trigger of the disease based on the consistent correlation of increased PAP and PVR with the severity of hypoxemia in COPD patients [58]–[60]. At rest, PH associated with COPD commonly causes only modest hemodynamic alterations in the pulmonary circulation as compared to other forms of PH [17]. Yet, mean PAP is a good predictor of exercise capacity [15], [61] and a better prognostic factor than the forced expiratory volume in the first second (FEV_1_) or the degrees of hypoxemia or hypercapnia in COPD patients [62]. Over time, PH secondary to COPD tends to progress with a reported rise in mean PAP of 0.5–1.5 mmHg per year [63], [64], and may ultimately cause exercise intolerance [65], [66], right ventricular dysfunction [67] and thus, potentially contribute to patient mortality in COPD [68]. While it is tempting to hypothesize that these associations of PH with clinical outcome may reflect a significant clinical relevance of PH in COPD, recent clinical trials targeting the pulmonary vasculature in patients with COPD and PH seemingly point to the contrary. Systemic delivery of the dual endothelin receptor antagonist bosentan or the phosphodiesterase 5 inhibitor sildenafil not only failed to improve exercise capacity [20]–[22], stroke volume [21], or quality of life [20], but also deteriorated arterial hypoxemia and functional status in patients with severe COPD [22]. These adverse effects are likely attributable to a non-specific inhibition of HPV by systemically delivered vasodilators in a heterogeneously ventilated lung resulting in impaired ventilation/perfusion matching and reduced oxygenation [69]. This notion is substantiated by the finding that sildenafil treatment in COPD patients with PH tends to divert blood flow to units with a low ventilation/perfusion ratio of less than one [19].

Inhaled delivery of vasodilators may potentially circumvent this critical pitfall, in that it restricts the pharmacological effect to ventilated and aerated lung regions, provided the inhaled drug does not spill over into the circulation in relevant amounts. Notably, one of the most effective drugs to attenuate HPV specifically in ventilated lung areas, namely supplemental oxygen, is already commonly used in patients with severe COPD and hypoxemia with or without cor pulmonale. However, oxygenation typically remains suboptimal and can be further improved by additional strategies such as non-invasive positive pressure ventilation [70] or inhalation of nitric oxide [71].

Consistent with a potential advantage of inhaled vasodilator delivery, inhalation of the stable prostacyclin analog iloprost has been reported to achieve short-term improvements in exercise capacity in the absence of adverse effects on arterial oxygenation or ventilation/perfusion matching in COPD patients in two small clinical trials [72], [73], and allowed for long-term improvement of pulmonary hemodynamics and exercise tolerance over a 2 year period in a single case of COPD with severe PH [74]. Conversely however, a recent crossover study in 16 COPD patients with confirmed PH failed to detect an improvement in six minute walk test following an acute inhalation of iloprost while oxygenation at rest significantly decreased [75]. Notably, inhaled doses of 10 and 20 µg iloprost in the latter "negative" study exceeded the individual doses of 2.5 and 5 µg in the previous, "positive" reports [73], [74], raising the possibility that systemic spillover of the inhaled drug may have caused or contributed to the observed adverse effects.

While the final verdict on the therapeutic benefit (or harm) of vasodilator treatment in COPD with PH is hence still pending, inhaled drug delivery with minimal systemic spillover would seem to present the most targeted and thus, promising approach. In the present study, we therefore tested the pulmonary vasodilatory potential of RO 25-1553 in the isolated perfused mouse lung as basic proof-of-principle, and upon inhalation in rats *in vivo*. In isolated lungs, RO 25-1553 had a dose-dependent inhibitory effect on HPV. As the pulmonary circulation is almost completely dilated under resting conditions [26], [27], RO 25-1553 (and likewise, the positive control sildenafil) did not reduce PAP at normoxia in isolated murine lungs. However, calculation of intrinsic vascular resistance which yields a more robust, pressure-independent measure of vascular tone, also revealed a direct vasodilatory potential of RO 25-1553 in non-preconstricted lungs. *In vivo*, inhalation of RO 25-1553 largely attenuated the hypoxia-induced increase in PAP, yet without affecting cardiac output, and thus effectively inhibited the increase in PVR. Importantly, this fall in PAP and PVR in response to RO 25-1553 inhalation was not associated with a decrease in systemic arterial pressure (AP) or an increased pulmonary shunt fraction (*Qs*/*Qt*). These findings indicate that the drug acted specifically on the pulmonary blood vessels without spillover into the systemic circulation or impairment of ventilation/perfusion matching. Inhaled delivery of RO 25-1553 may therefore potentially circumvent some of the problems inherent to the systemic delivery of vasodilators in previous COPD trials.

Of interest, hypoxic ventilation by itself increased the intrapulmonary shunt fraction and caused mild-to-moderate edema formation in the present *in vivo* experiments as signified by a small increase in the wet-to-dry lung weight ratio. Notably, the estimation of CcO_2_ for the calculation of *Qs*/*Qt* according to the classic shunt equation is based on the assumption of 100% capillary oxygen saturation, which is clearly not the case in hypoxic ventilation. As a result, the absolute increase of *Qs*/*Qt* in response to hypoxia as given in Fig. 3C is probably inflated; however, as this systematic constraint applies equally to all hypoxic groups, it does not affect the intergroup comparison between hypoxic animals inhaling either NaCl, RO 25-1553, or sildenafil. In elegant human studies, Lovering and coworkers had previously demonstrated that hypoxia induces intrapulmonary arteriovenous shunt pathways [33], yet whether oxygen tension specifically regulates these novel pathways or opens them indirectly via effects on the conventional pulmonary vasculature remained so far unclear. Our present finding that both inhaled RO 25-1553 and sildenafil attenuated the increases in PAP and PVR without affecting the hypoxia-induced increase in *Qs*/*Qt* suggests that opening of intrapulmonary shunts occurs independent of HPV and thus, changes in hydrostatic forces. Likewise, the formation of hypoxic pulmonary edema which had previously been described in both resting and exercising rats [34], was apparently not attributable to increased hydrodynamic forces subsequent to HPV, but rather to hypoxia-induced increases in lung microvascular permeability and/or inhibition of alveolar fluid absorption as previously proposed [76], [77].

While the present findings suggest that inhaled RO 25-1553 may strike an attractive balance between beneficial efficiency in terms of improved pulmonary hemodynamics and absence of adverse effects on ventilation/perfusion mismatching, lung edema formation, and systemic hemodynamics in hypoxic lung disease, caution is clearly warranted not only in light of the recent negative results with aerosolized iloprost in COPD-related PH [75]. Inhaled vasodilators have also been proposed to promote edema formation in patients with left heart dysfunction, a common comorbidity in COPD, by inhibition of the so-called Kitajew reflex [78]. Finally, we should remind ourselves that the present findings were obtained in experimental models of acute hypoxia, and similar efficacy and safety in clinically more relevant chronic models of COPD and ultimately, clinical trials remains to be shown.

## References

[1] Viegi G, Pistelli F, Sherrill DL, Maio S, Baldacci S, et al (2007) Definition, epidemiology and natural history of COPD. Eur Respir J 30: 993–1013. 17978157 10.1183/09031936.00082507

[2] Said SI, Mutt V (1970) Polypeptide with broad biological activity: isolation from small intestine. Science 169: 1217–1218. 5450698 10.1126/science.169.3951.1217

[3] Onoue S, Misaka S, Yamada S (2008) Structure-activity relationship of vasoactive intestinal peptide (VIP): potent agonists and potential clinical applications. Naunyn Schmiedebergs Arch Pharmacol 377: 579–590. 18172612 10.1007/s00210-007-0232-0

[4] Dey RD, Shannon WA Jr, Said SI (1981) Localization of VIP-immunoreactive nerves in airways and pulmonary vessels of dogs, cat, and human subjects. Cell Tissue Res 220: 231–238. 7296630 10.1007/BF00210505

[5] Lundberg JM, Fahrenkrug J, Hokfelt T, Martling CR, Larsson O, et al (1984) Co-existence of peptide HI (PHI) and VIP in nerves regulating blood flow and bronchial smooth muscle tone in various mammals including man. Peptides 5: 593–606. 6382200 10.1016/0196-9781(84)90090-1

[6] Laburthe M, Couvineau A, Marie JC (2002) VPAC receptors for VIP and PACAP. Receptors Channels 8: 137–153. 12529932

[7] Usdin TB, Bonner TI, Mezey E (1994) Two receptors for vasoactive intestinal polypeptide with similar specificity and complementary distributions. Endocrinology 135: 2662–2680. 7988457 10.1210/endo.135.6.7988457

[8] Busto R, Prieto JC, Bodega G, Zapatero J, Carrero I (2000) Immunohistochemical localization and distribution of VIP/PACAP receptors in human lung. Peptides 21: 265–269. 10764955 10.1016/s0196-9781(99)00202-8

[9] O'Donnell M, Garippa RJ, Rinaldi N, Selig WM, Simko B, et al (1994) Ro 25-1553: a novel, long-acting vasoactive intestinal peptide agonist. Part I: In vitro and in vivo bronchodilator studies. J Pharmacol Exp Ther 270: 1282–1288. 7932180

[10] O'Donnell M, Garippa RJ, Rinaldi N, Selig WM, Tocker JE, et al (1994) Ro 25-1553: a novel, long-acting vasoactive intestinal peptide agonist. Part II: Effect on in vitro and in vivo models of pulmonary anaphylaxis. J Pharmacol Exp Ther 270: 1289–1294. 7932181

[11] Tannu SA, Renzetti LM, Tare N, Ventre JD, Lavelle D, et al (2010) Dual bronchodilatory and pulmonary anti-inflammatory activity of RO5024118, a novel agonist at vasoactive intestinal peptide VPAC2 receptors. Br J Pharmacol 161: 1329–1342. 20735404 10.1111/j.1476-5381.2010.00975.x PMC3000657

[12] Said SI (2006) Mediators and modulators of pulmonary arterial hypertension. Am J Physiol Lung Cell Mol Physiol 291: L547–L558. 16698850 10.1152/ajplung.00546.2005

[13] Said SI (2008) The vasoactive intestinal peptide gene is a key modulator of pulmonary vascular remodeling and inflammation. Ann N Y Acad Sci 1144: 148–153. 19076374 10.1196/annals.1418.014

[14] Minkes RK, McMahon TJ, Higuera TR, Murphy WA, Coy DH, et al (1992) Analysis of systemic and pulmonary vascular responses to PACAP and VIP: role of adrenal catecholamines. Am J Physiol 263: H1659–H1669. 1481892 10.1152/ajpheart.1992.263.6.H1659

[15] Cuttica MJ, Kalhan R, Shlobin OA, Ahmad S, Gladwin M, et al (2010) Categorization and impact of pulmonary hypertension in patients with advanced COPD. Respir Med 104: 1877–1882. 20547449 10.1016/j.rmed.2010.05.009

[16] Thabut G, Dauriat G, Stern JB, Logeart D, Levy A, et al (2005) Pulmonary hemodynamics in advanced COPD candidates for lung volume reduction surgery or lung transplantation. Chest 127: 1531–1536. 15888824 10.1378/chest.127.5.1531

[17] Minai OA, Chaouat A, Adnot S (2010) Pulmonary hypertension in COPD: epidemiology, significance, and management: pulmonary vascular disease: the global perspective. Chest 137: 39S–51S. 20522579 10.1378/chest.10-0087

[18] Hurdman J, Condliffe R, Elliot CA, Swift A, Rajaram S, et al (2013) Pulmonary hypertension in COPD: results from the ASPIRE registry. Eur Respir J 41: 1292–1301. 23018917 10.1183/09031936.00079512

[19] Blanco I, Gimeno E, Munoz PA, Pizarro S, Gistau C, et al (2010) Hemodynamic and gas exchange effects of sildenafil in patients with chronic obstructive pulmonary disease and pulmonary hypertension. Am J Respir Crit Care Med 181: 270–278. 19875684 10.1164/rccm.200907-0988OC

[20] Blanco I, Santos S, Gea J, Guell R, Torres F, et al (2013) Sildenafil to improve respiratory rehabilitation outcomes in COPD: a controlled trial. Eur Respir J [Epub ahead of print] 10.1183/09031936.00176312 23429918

[21] Rietema H, Holverda S, Bogaard HJ, Marcus JT, Smit HJ, et al (2008) Sildenafil treatment in COPD does not affect stroke volume or exercise capacity. Eur Respir J 31: 759–764. 18094009 10.1183/09031936.00114207

[22] Stolz D, Rasch H, Linka A, Di Valentino M, Meyer A, et al (2008) A randomised, controlled trial of bosentan in severe COPD. Eur Respir J 32: 619–628. 18448495 10.1183/09031936.00011308

[23] Urban N, Hill K, Wang L, Kuebler WM, Schaefer M (2012) Novel pharmacological TRPC inhibitors block hypoxia-induced vasoconstriction. Cell Calcium 51: 194–206. 22280812 10.1016/j.ceca.2012.01.001

[24] Spohr F, Busch CJ, Reich C, Motsch J, Gebhard MM, et al (2007) 4-Aminopyridine restores impaired hypoxic pulmonary vasoconstriction in endotoxemic mice. Anesthesiology 107: 597–604. 17893456 10.1097/01.anes.0000281897.13703.fd

[25] Linehan JH, Haworth ST, Nelin LD, Krenz GS, Dawson CA (1992) A simple distensible vessel model for interpreting pulmonary vascular pressure-flow curves. J Appl Physiol 73: 987–94. 1400067 10.1152/jappl.1992.73.3.987

[26] Hentschel T, Yin N, Riad A, Habbazettl H, Weimann J, et al (2007) Inhalation of the phosphodiesterase-3 inhibitor milrinone attenuates pulmonary hypertension in a rat model of congestive heart failure. Anesthesiology 106: 124–131. 17197854 10.1097/00000542-200701000-00021

[27] Yin N, Kaestle S, Yin J, Hentschel T, Pries AR, et al (2009) Inhaled nitric oxide versus aerosolized iloprost for the treatment of pulmonary hypertension with left heart disease. Crit Care Med 37: 980–986. 19237907 10.1097/CCM.0b013e3181962ce6

[28] Tabuchi A, Mertens M, Kuppe H, Pries AR, Kuebler WM (2008) Intravital microscopy of the murine pulmonary microcirculation. J Appl Physiol 104: 338–346. 18006870 10.1152/japplphysiol.00348.2007

[29] Fenn WO, Rahn H, Otis AB (1946) A theoretical study of the composition of the alveolar air at altitude. Am J Physiol 146: 637–653. 20996488 10.1152/ajplegacy.1946.146.5.637

[30] Peterson BT, Brooks JA, Zack AG (1982) Use of microwave oven for determination of postmortem water volume of lungs. J Appl Physiol 52: 1661–1663. 7107478 10.1152/jappl.1982.52.6.1661

[31] Zhao L, Mason NA, Morrell NW, Kojonazarov B, Sadykov A, et al (2001) Sildenafil inhibits hypoxia-induced pulmonary hypertension. Circulation 104: 424–428. 11468204 10.1161/hc2901.093117

[32] Ichinose F, Erana-Garcia J, Hromi J, Raveh Y, Jones R, et al (2001) Nebulized sildenafil is a selective pulmonary vasodilator in lambs with acute pulmonary hypertension. Crit Care Med 29: 1000–1005. 11378612 10.1097/00003246-200105000-00024

[33] Lovering AT, Romer LM, Haverkamp HC, Pegelow DF, Hokanson JS, et al (2008) Intrapulmonary shunting and pulmonary gas exchange during normoxic and hypoxic exercise in healthy humans. J Appl Physiol 104: 1418–1425. 18292301 10.1152/japplphysiol.00208.2007

[34] Whayne TF Jr, Severinghaus JW (1968) Experimental hypoxic pulmonary edema in the rat. J Appl Physiol 25: 729–732. 5727200 10.1152/jappl.1968.25.6.729

[35] Said SI (1991) Vasoactive intestinal polypeptide (VIP) in asthma. Ann N Y Acad Sci 629: 305–318. 1683200 10.1111/j.1749-6632.1991.tb37985.x

[36] Onoue S, Yamada S, Yajima T (2007) Bioactive analogues and drug delivery systems of vasoactive intestinal peptide (VIP) for the treatment of asthma/COPD. Peptides 28: 1640–1650. 17537541 10.1016/j.peptides.2007.04.009

[37] Groneberg DA, Rabe KF, Fischer A (2006) Novel concepts of neuropeptide-based drug therapy: vasoactive intestinal polypeptide and its receptors. Eur J Pharmacol 533: 182–194. 16473346 10.1016/j.ejphar.2005.12.055

[38] Ghatei MA, Sheppard MN, O'Shaughnessy DJ, Adrian TE, McGregor GP, et al (1982) Regulatory peptides in the mammalian respiratory tract. Endocrinology 111: 1248–1254. 6180890 10.1210/endo-111-4-1248

[39] Paul S, Said SI (1987) Characterization of receptors for vasoactive intestinal peptide solubilized from the lung. J Biol Chem 262: 158–162. 3025200

[40] Morice A, Unwin RJ, Sever PS (1983) Vasoactive intestinal peptide causes bronchodilatation and protects against histamine-induced bronchoconstriction in asthmatic subjects. Lancet 2: 1225–1227. 6139572 10.1016/s0140-6736(83)91272-2

[41] Domschke S, Domschke W, Bloom SR, Mitznegg P, Mitchell SJ, et al (1978) Vasoactive intestinal peptide in man: pharmacokinetics, metabolic and circulatory effects. Gut 19: 1049–1053. 730072 10.1136/gut.19.11.1049 PMC1412244

[42] Hassan M, Refai E, Andersson M, Schnell PO, Jacobsson H (1994) In vivo dynamical distribution of ^131^I-VIP in the rat studied by γ-camera. Nucl Med Biol 21: 865–872. 9234336 10.1016/0969-8051(94)90166-x

[43] Bolin DR, Michalewsky J, Wasserman MA, O'Donnell M (1995) Design and development of a vasoactive intestinal peptide analog as a novel therapeutic for bronchial asthma. Biopolymers 37: 57–66. 7893947 10.1002/bip.360370203

[44] Dewit D, Gourlet P, Amraoui Z, Vertongen P, Willems F, et al (1998) The vasoactive intestinal peptide analogue RO25-1553 inhibits the production of TNF and IL-12 by LPS-activated monocytes. Immunol Lett 60: 57–60. 9541464 10.1016/s0165-2478(97)00129-6

[45] Kallstrom BL, Waldeck B (2001) Bronchodilating properties of the VIP receptor agonist Ro 25-1553 compared to those of formoterol on the guinea-pig isolated trachea. Eur J Pharmacol 430: 335–340. 11711052 10.1016/s0014-2999(01)01299-7

[46] Linden A, Hansson L, Andersson A, Palmqvist M, Arvidsson P, et al (2003) Bronchodilation by an inhaled VPAC_2_ receptor agonist in patients with stable asthma. Thorax 58: 217–221. 12612296 10.1136/thorax.58.3.217 PMC1746614

[47] Szema AM, Hamidi SA, Lyubsky S, Dickman KG, Mathew S, et al (2006) Mice lacking the VIP gene show airway hyperresponsiveness and airway inflammation, partially reversible by VIP. Am J Physiol Lung Cell Mol Physiol 291: L880–L886. 16782752 10.1152/ajplung.00499.2005

[48] Said SI, Hamidi SA, Dickman KG, Szema AM, Lyubsky S, et al (2007) Moderate pulmonary arterial hypertension in male mice lacking the vasoactive intestinal peptide gene. Circulation 115: 1260–1268. 17309917 10.1161/CIRCULATIONAHA.106.681718

[49] Saga T, Said SI (1984) Vasoactive intestinal peptide relaxes isolated strips of human bronchus, pulmonary artery, and lung parenchyma. Trans Assoc Am Physicians 97: 304–310. 6535346

[50] Hamasaki Y, Saga T, Mojarad M, Said SI (1983) Vasoactive intestinal peptide counteracts leukotriene D_4_-induced contractions of guinea pig trachea, lung, and pulmonary artery. Trans Assoc Am Physicians 96: 406–411. 6679961

[51] Nandiwada PA, Kadowitz PJ, Said SI, Mojarad M, Hyman AL (1985) Pulmonary vasodilator responses to vasoactive intestinal peptide in the cat. J Appl Physiol 58: 1723–1728. 3997734 10.1152/jappl.1985.58.5.1723

[52] Schmidt DT, Ruhlmann E, Waldeck B, Branscheid D, Luts A, et al (2001) The effect of the vasoactive intestinal polypeptide agonist Ro 25-1553 on induced tone in isolated human airways and pulmonary artery. Naunyn Schmiedebergs Arch Pharmacol 364: 314–320. 11683518 10.1007/s002100100458

[53] Leuchte HH, Baezner C, Baumgartner RA, Bevec D, Bacher G, et al (2008) Inhalation of vasoactive intestinal peptide in pulmonary hypertension. Eur Respir J 32: 1289–1294. 18978135 10.1183/09031936.00050008

[54] Petkov V, Mosgoeller W, Ziesche R, Raderer M, Stiebellehner L, et al (2003) Vasoactive intestinal peptide as a new drug for treatment of primary pulmonary hypertension. J Clin Invest 111: 1339–1346. 12727925 10.1172/JCI17500 PMC154449

[55] Galie N, Boonstra A, Ewert R, Gomez-Sanchez MA, Barbera JA, , et al (2010) Effects of inhaled aviptadil (vasoactive intestinal peptide) in patients with pulmonary arterial hypertension (PAH). Am J Respir Crit Care Med 181: A2516 [Abstract]

[56] Said SI (2012) Vasoactive intestinal peptide in pulmonary arterial hypertension. Am J Respir Crit Care Med 185: 786. 10.1164/ajrccm.185.7.786 22467806

[57] Simonneau G, Robbins IM, Beghetti M, Channick RN, Delcroix M, et al (2009) Updated clinical classification of pulmonary hypertension. J Am Coll Cardiol 54: S43–S54. 19555858 10.1016/j.jacc.2009.04.012

[58] Chatila WM, Thomashow BM, Minai OA, Criner GJ, Make BJ (2008) Comorbidities in chronic obstructive pulmonary disease. Proc Am Thorac Soc 5: 549–55. 18453370 10.1513/pats.200709-148ET PMC2645334

[59] Falk JA, Kadiev S, Criner GJ, Scharf SM, Minai OA, et al (2008) Cardiac disease in chronic obstructive pulmonary disease. Proc Am Thorac Soc 5: 543–8. 18453369 10.1513/pats.200708-142ET PMC2645333

[60] Chaouat A, Naeije R, Weitzenblum E (2008) Pulmonary hypertension in COPD. Eur Respir J 32: 1371–1385. 18978137 10.1183/09031936.00015608

[61] Sims MW, Margolis DJ, Localio AR, Panettieri RA, Kawut SM, et al (2009) Impact of pulmonary artery pressure on exercise function in severe COPD. Chest 136: 412–419. 19318664 10.1378/chest.08-2739 PMC2818413

[62] Oswald-Mammosser M, Weitzenblum E, Quoix E, Moser G, Chaouat A, et al (1995) Prognostic factors in COPD patients receiving long-term oxygen therapy. Importance of pulmonary artery pressure. Chest 107: 1193–1198. 7750305 10.1378/chest.107.5.1193

[63] Kessler R, Faller M, Weitzenblum E, Chaouat A, Aykut A, et al (2001) "Natural history" of pulmonary hypertension in a series of 131 patients with chronic obstructive lung disease. Am J Respir Crit Care Med 164: 219–224. 11463591 10.1164/ajrccm.164.2.2006129

[64] Weitzenblum E, Sautegeau A, Ehrhart M, Mammosser M, Pelletier A (1985) Long-term oxygen therapy can reverse the progression of pulmonary hypertension in patients with chronic obstructive pulmonary disease. Am Rev Respir Dis 131: 493–498. 3922267 10.1164/arrd.1985.131.4.493

[65] Oswald-Mammosser M, Apprill M, Bachez P, Ehrhart M, Weitzenblum E (1991) Pulmonary hemodynamics in chronic obstructive pulmonary disease of the emphysematous type. Respiration 58: 304–310. 1792422 10.1159/000195950

[66] Christensen CC, Ryg MS, Edvardsen A, Skjonsberg OH (2004) Relationship between exercise desaturation and pulmonary haemodynamics in COPD patients. Eur Respir J 24: 580–586. 15459136 10.1183/09031936.04.00118303

[67] Vizza CD, Lynch JP, Ochoa LL, Richardson G, Trulock EP (1998) Right and left ventricular dysfunction in patients with severe pulmonary disease. Chest 113: 576–583. 9515827 10.1378/chest.113.3.576

[68] Burrows B, Kettel LJ, Niden AH, Rabinowitz M, Diener CF (1972) Patterns of cardiovascular dysfunction in chronic obstructive lung disease. N Engl J Med 286: 912–918. 5013974 10.1056/NEJM197204272861703

[69] Bull T, Badesch DB (2012) Sildenafil for COPD: a randomized crossover trial. COPD 9: 211–212. 22587530 10.3109/15412555.2012.683966

[70] Dreher M, Doncheva E, Schwoerer A, Walterspacher S, Sonntag F, et al (2009) Preserving oxygenation during walking in severe chronic obstructive pulmonary disease: noninvasive ventilation versus oxygen therapy. Respiration 78: 154–160. 19092234 10.1159/000187717

[71] Yoshida M, Taguchi O, Gabazza EC, Kobayashi T, Yamakami T, et al (1997) Combined inhalation of nitric oxide and oxygen in chronic obstructive pulmonary disease. Am J Respir Crit Care Med 155: 526–529. 9032189 10.1164/ajrccm.155.2.9032189

[72] Marino WD, Granville P, (2006) The effect of iloprost inhalation on gas exchange in patients with COPD. Chest 130: 178S–179S [Abstract]

[73] Dernaika TA, Beavin M, Kinasewitz GT (2010) Iloprost improves gas exchange and exercise tolerance in patients with pulmonary hypertension and chronic obstructive pulmonary disease. Respiration 79: 377–382. 19786728 10.1159/000242498

[74] Hegewald MJ, Elliott CG (2009) Sustained improvement with iloprost in a COPD patient with severe pulmonary hypertension. Chest 135: 536–537. 19201716 10.1378/chest.08-1515

[75] Boeck L, Tamm M, Grendelmeier P, Stolz D (2012) Acute effects of aerosolized iloprost in COPD related pulmonary hypertension - a randomized controlled crossover trial. PLoS One 7: e52248. 23300624 10.1371/journal.pone.0052248 PMC3531427

[76] Dehler M, Zessin E, Bartsch P, Mairbaurl H (2006) Hypoxia causes permeability oedema in the constant-pressure perfused rat lung. Eur Respir J 27: 600–606. 16507862 10.1183/09031936.06.00061505

[77] Vivona ML, Matthay M, Chabaud MB, Friedlander G, Clerici C (2001) Hypoxia reduces alveolar epithelial sodium and fluid transport in rats: reversal by β-adrenergic agonist treatment. Am J Respir Cell Mol Biol 25: 554–561. 11713096 10.1165/ajrcmb.25.5.4420

[78] Olschewski H, Olschewski A, Rose F, Schermuly R, Schutte H, et al (2001) Physiologic basis for the treatment of pulmonary hypertension. J Lab Clin Med 138: 287–297. 11709653 10.1067/mlc.2001.119329

